# Establishment of a dual-wavelength spectrophotometric method for analysing and detecting carbapenemase-producing *Enterobacteriaceae*

**DOI:** 10.1038/s41598-018-33883-0

**Published:** 2018-10-24

**Authors:** Dan Takeuchi, Yukihiro Akeda, Yo Sugawara, Noriko Sakamoto, Norihisa Yamamoto, Rathina Kumar Shanmugakani, Takuma Ishihara, Ayumi Shintani, Kazunori Tomono, Shigeyuki Hamada

**Affiliations:** 1 Section of Bacterial Drug Resistance Research, Japan-Thailand Research Collaboration Center on Emerging and Re-emerging Infections, Research Institute for Microbial Diseases, Osaka University, Osaka, Japan; 2 Division of Infection Control and Prevention, Graduate School of Medicine, Osaka University, Osaka, Japan; 3 Department of Medical Statistics, Osaka City University Graduate School of Medicine, Osaka, Japan

## Abstract

The spread of carbapenemase-producing *Enterobacteriaceae* (CPE) is an increasing global public health concern. The development of simple and reliable methods for CPE detection is required in the clinical setting. This study aimed to establish a dual-wavelength measurement method using an ultraviolet–visible spectrophotometer to rapidly quantify imipenem hydrolysis in bacterial cell suspensions. The hydrolytic activities of 148 strains including various CPE strains (*Escherichia coli*, *Klebsiella pneumoniae*, *Enterobacter cloacae*, and *Enterobacter aerogenes* containing the *bla*_IMP_, *bla*_KPC_, *bla*_NDM_, *bla*_OXA_, and *bla*_VIM_ genes) were measured and analysed. A cut-off value was obtained for differentiation between CPE and non-CPE strains, and the method had high sensitivity (100%) and specificity (100%) within 60 min. Our system has potential clinical applications in detecting CPE.

## Introduction

Carbapenemase-producing *Enterobacteriaceae* (CPE) have been increasingly reported worldwide in recent years; they frequently display multi-drug resistance and cause severe infections in immunocompromised patients, with limited treatment options. Rapid diagnosis of a CPE infection is required in the clinical setting for prognostic assessment, appropriate treatment, and infection-control measures^1–3^.

Several diagnostic tests have been developed to detect CPE^1,3^; These are of two types: nucleic acid-based and non-nucleic acid-based tests. Nucleic acid-based tests include the polymerase chain reaction (PCR) and sequencing and are considered one of the most reliable methods for confirming the presence of CPE. Carbapenemase types are categorized into Ambler class A serine carbapenemase (such as KPC), class B metallo-β-lactamase (such as NDM, IMP, and VIM), class D OXA-type carbapenemase (such as OXA-48), and their variants^4^. One of the advantages of nucleic-acid based tests is the capability of identifying the carbapenemase-type. However, high cost and the requirement of significant expertise frequently deter the implementation of nucleic acid tests in clinical microbiology laboratories.

Non-nucleic acid-based tests commonly harness the hydrolytic activity of carbapenemases. The Carba NP test is among the most widely used rapid detection tests^5–9^. The Carba NP test depends on imipenem hydrolysis by a carbapenemase and a subsequent change in pH and colour of Phenol Red in the reaction mixture. The colour change is discerned with the naked eye within 120 min, and the method is efficient with KPC-type carbapenemase and metallo-β-lactamase producers. However, the Carba NP test has shown lower detection sensitivity with OXA-type carbapenemase producers^6,7,9^.

Ultraviolet–visible (UV–VIS) spectrophotometry has been used to assess the hydrolytic activity of carbapenemases^7,10–15^. Imipenem displays maximum absorption at approximately 300 nm, and the absorbance decreases upon hydrolysis. Although this method has shown high sensitivity and specificity for detecting CPE, carbapenemase extraction from bacterial cells is somewhat time consuming and is not suitable for rapid detection.

In this study, we devised a new method to directly measure carbapenemase activity in bacterial cells via UV–visible spectrophotometry. Our method facilitates simple and rapid CPE detection with high sensitivity and specificity.

## Results

### Spectrophotometric analysis of imipenem

**Figure 1:**
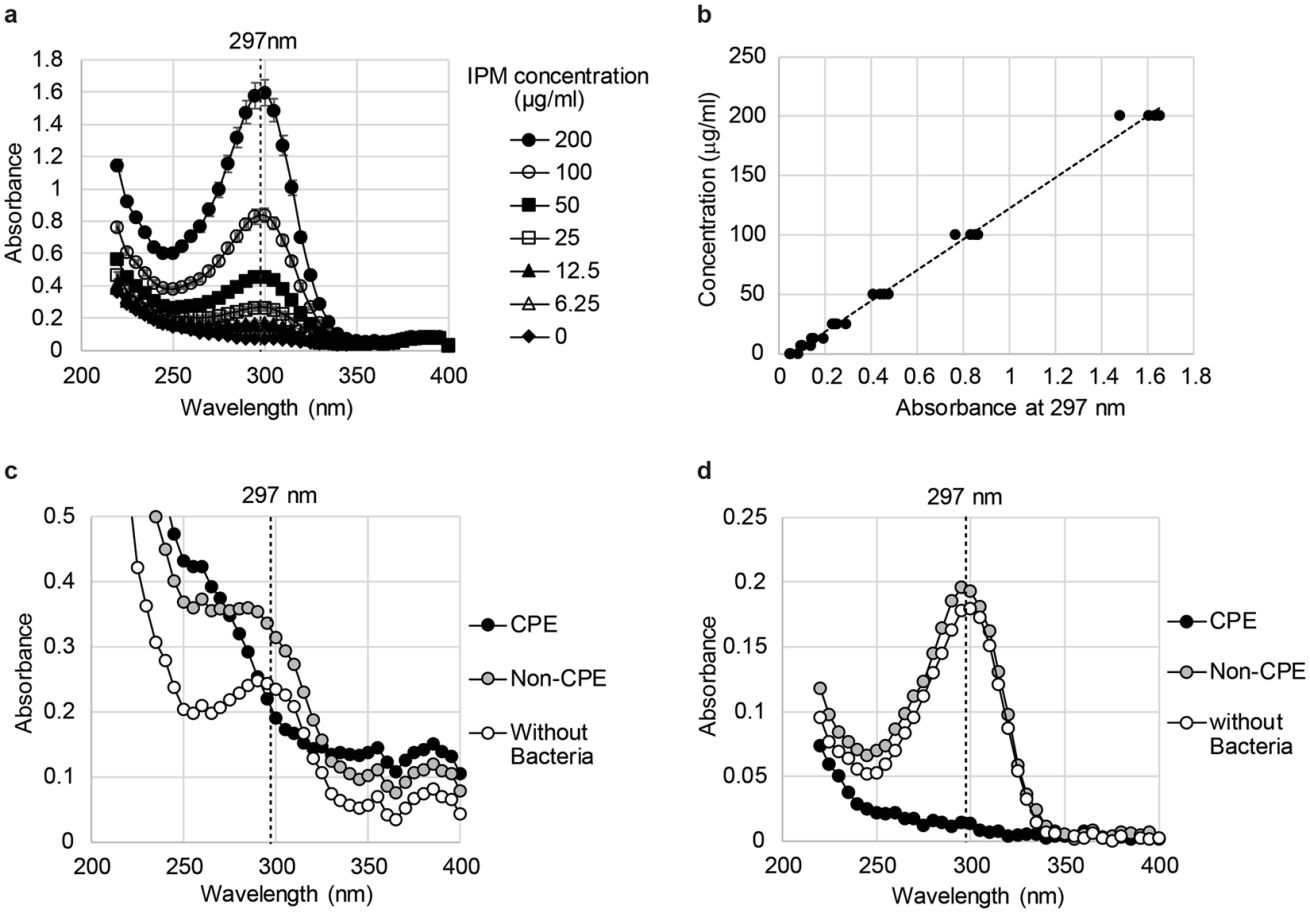
(**a**) Absorption spectra of imipenem (IPM) between 220 nm and 400 nm at different concentrations. Symbols show the mean of four independent measurements, and bars show standard deviations. (**b**) Relationship between the imipenem concentration and the absorbance at 297 nm. Curve fitting with linear-regression analysis was performed (r^2^ = 0.9956, β1 = 130.0076 [126.5346, 133.4806]), and the obtained curve is shown as a dotted line. Absorbance measurements were independently performed four times. Each symbol shows a measurement result. (**c**) Spectral analysis of imipenem hydrolysis before background subtraction. (**d**) Spectral analysis of imipenem hydrolysis after background subtraction. CPE: imipenem solution incubated with a carbapenemase-producing *Enterobacteriaceae* (CPE) strain, non-CPE: imipenem solution incubated with a non-CPE strains, Without Bacteria: imipenem solution without bacteria.

The absorption spectrum of imipenem in phosphate-buffered saline (PBS) was examined within the wavelength range of 220 nm to 400 nm. Similar to a previous report^10^, the absorption maxima for imipenem was between 250 nm and 350 nm, with an absorption peak at 297 nm (Fig. 1a). The absorbance of imipenem at 297 nm correlated linearly with imipenem concentrations of up to 200 μg/ml, which facilitated the spectrophotometric estimation of the imipenem concentration (Fig. 1b).

### Imipenem hydrolysis by bacterial cells

Enzymatic hydrolysis of imipenem by CPE and non-CPE strains was examined by measuring the absorbance of bacterial cell suspensions. It was difficult to directly compare the gross absorbance of CPE and non-CPE suspensions at 297 nm because of differences in the baseline absorbance spectra of each bacterial type (Fig. 1c). To calculate the net imipenem absorbance, we subtracted the background absorbance from the gross absorbance, i.e. the absorbance of a bacterial suspension without imipenem was subtracted from that of the bacterial suspension with imipenem (Supplementary Fig. S1). Imipenem hydrolysis in CPE suspensions was clearly demonstrated by the disappearance of the characteristic absorption peak of imipenem, whereas the peak in non-CPE suspension hardly changed (Fig. 1d).

### Estimation of background absorbance

**Figure 2:**
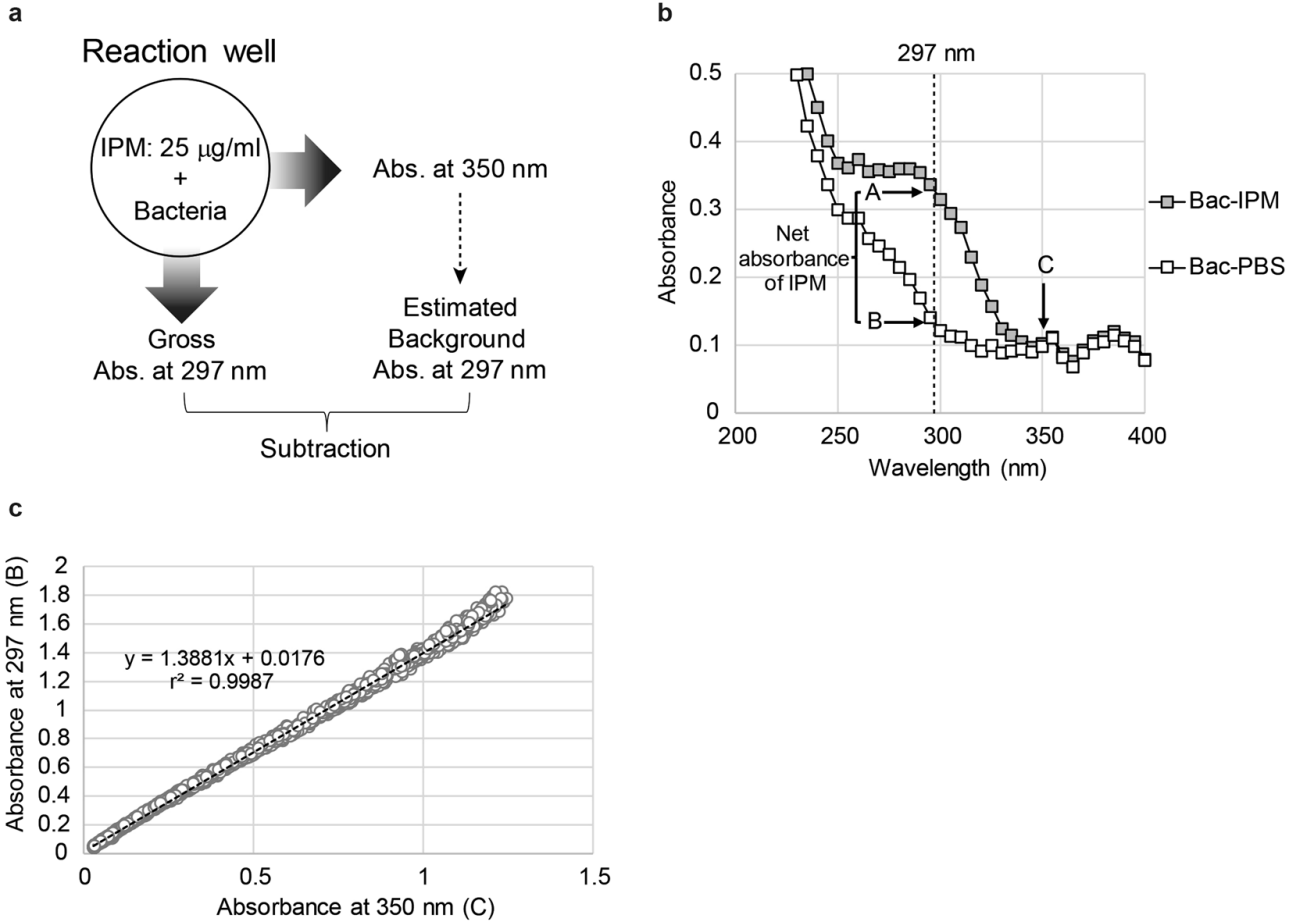
(**a**) A schematic representation of sample preparation for dual-wavelength spectrophotometric measurement. Instead of preparing a background well, the absorbance at 297 nm and 350 nm are measured simultaneously using a single reaction well to calculate the estimated net absorbance of imipenem. IPM = imipenem, Abs. = absorbance. (**b**) Spectral analysis of bacterial suspensions incubated with (Bac-IPM) or without (Bac-PBS) imipenem. The absorbances at 297 nm of Bac-IPM and Bac-PBS are denoted as A and B, which correspond to the gross absorbance of imipenem and background absorbance, respectively. The absorbance of both suspensions at 350 nm is denoted as C. (**c**) Relationship between points B and C. The absorbances of 15 representative strains were independently measured six times, at eight different concentrations and after four different incubation times. Curve fitting with linear-regression analysis was performed, and the obtained curve is shown as a dotted line with equation (1) and r^2^ value. The statistical values are summarized in Supplementary Table S2.

The preparation of separate bacterial suspensions for individual background subtractions is time-consuming, and unequal bacterial densities in the reaction and background wells may result in inaccurate background correction. In the interest of maximizing the utility of our assay system, it was preferable to obtain background data from reaction wells (Fig. 2a). The net absorbance of imipenem at 297 nm was designated as the residual absorbance after subtracting data point B (without imipenem) from data point A (with imipenem), as shown in Fig. 2b. Figure 2b also shows that the absorbances in the presence of imipenem or PBS were closely matched at 350 nm (point C). Thus, a linear relationship was observed when plotting the absorbance of 15 representative strains (Supplementary Table S1) at 350 nm (point C) versus 297 nm (point B), with a r^2^ value of 0.9987 (Fig. 2c). The estimated background absorbance (eAbs.297 nm) was calculated from the approximation equation (1):

1 $${\rm{eAbs}}.{\rm{297}}\,{\rm{nm}}={\rm{1.3881}}\times {\rm{Abs}}.{\rm{350}}\,{\rm{nm}}+{\rm{0.0176}}$$

(Fig. 2c and Supplementary Table S2). Accordingly, the estimated net absorbance of imipenem could be obtained via dual-wavelength absorbance measurements at 297 nm and 350 nm using a single reaction mixture, followed by the subtraction of eAbs.297 nm from the gross absorbance at 297 nm (gAbs.297 nm).

The wavelength of 350 nm was selected because it represented the end of the absorption spectrum of imipenem, and the effect of imipenem on the absorbance of bacterial suspensions was statistically negligible (Supplementary Fig. S2). Furthermore, the linearity and fitness of equation (1) was best for the relationship between 297 nm and 350 nm (Fig. 2c), among the tested wavelengths (Supplementary Fig. S3).

### Quantification of bacterial hydrolytic activity to imipenem

**Figure 3:**
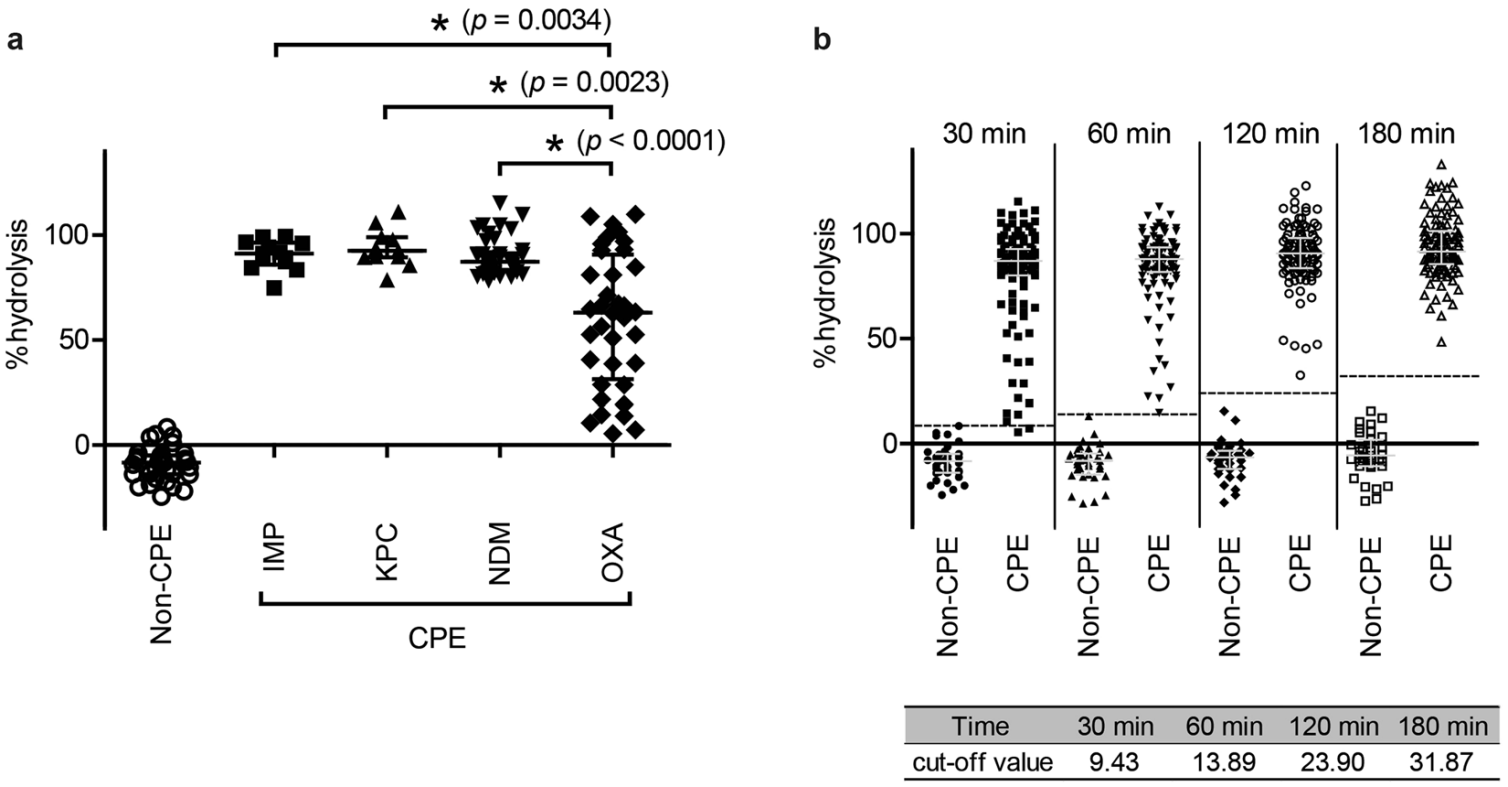
(**a**) The percent hydrolysis of carbapenemase-producing *Enterobacteriaceae* (CPE; n = 112) and non-CPE (n = 35) strains after a 30-min incubation. The hydrolytic activities were compared among four different kinds of carbapenemase producers, i.e. IMP (n = 13), KPC (n = 11), NDM (n = 52), and OXA-producers (n = 36). A VIM-type carbapenemase producer was not included in the analysis because of the small sample number. The symbols (open circles, black squares, black triangles, and black diamonds) show percent-hydrolysis results for each type of strain tested. The bars show the median with the interquartile range. The Kruskal–Wallis test was performed for global comparisons (*p* < 0.0001), followed by two-tailed Mann–Whitney testing. *Significantly different. (**b**) The percent-hydrolysis of CPE (n = 113) and non-CPE (n = 35) strains after the indicated incubation times. The symbols (black circles, black squares, black triangles, black diamonds, open circles, open squares, and open triangles) show the percent hydrolysis results for each type of strain tested. The bars show the median with the interquartile range. The horizontal dashed lines show the cut-off value for each incubation time. The cut-off values are also summarized at the bottom of the figure.

The hydrolytic activities of 148 strains (35 non-CPE strains and 113 CPE strains) were examined using dual-wavelength spectrophotometry, and the percent hydrolysis was calculated. OXA-type carbapenemase producers exhibited lower hydrolytic activities than the other four kinds of CPE strains after a 30-min incubation with imipenem (Fig. 3a). With increasing incubation times, percent hydrolysis by OXA producers gradually increased (Supplementary Fig. S4), and that of CPE strains was clearly distinct from that of non-CPE strains after a 120-min incubation (Fig. 3b).

**Figure 4:**
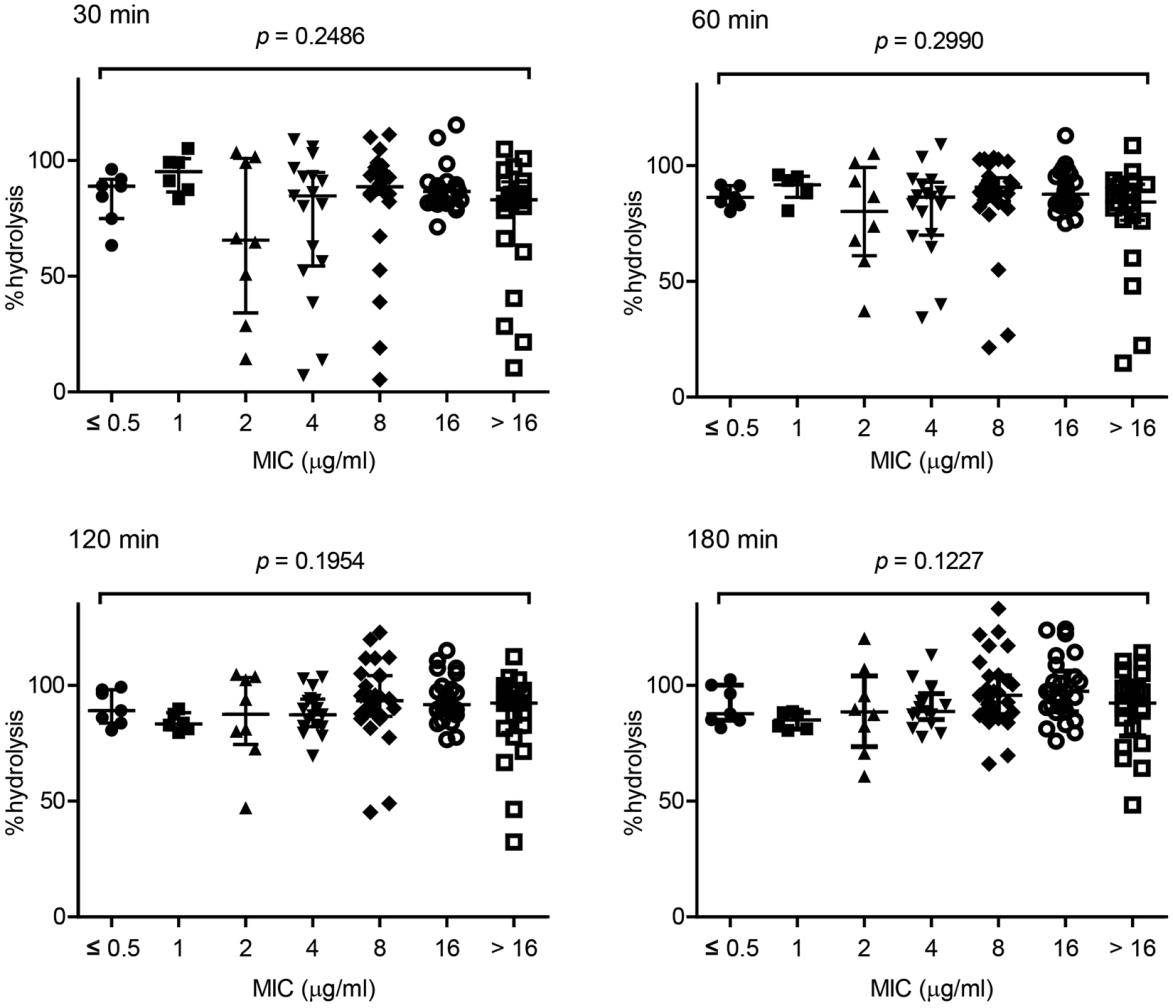
Statistically non-significant relationships between the minimal inhibitory concentrations (MICs) of imipenem and the percent hydrolysis of carbapenemase-producing *Enterobacteriaceae* (CPE) strains after incubation for 30, 60, 120, or 180 min. CPE strains (n = 113) were divided into seven groups based on the MICs of imipenem, i.e. ≤0.5 (n = 7), 1 (n = 6), 2 (n = 8), 4 (n = 17), 8 (n = 29), 16 (n = 25), and >16 (n = 21). The symbols (black circles, black squares, black triangles, black diamonds, open circles, and open squares) shows percent-hydrolysis results for each type of strain tested. The bars show the median with the interquartile range. Kruskal–Wallis testing was used for comparisons.

The minimal inhibitory concentrations (MICs) of imipenem for CPE strains were not reflected by their percent hydrolysis values, as no significant difference was noted among different groups classified in accordance with the MICs (Fig. 4). Strong hydrolytic activity was observed even for the CPE strains with low MICs ≤ 1.0 μg/ml, as with non-CPE strains. (Fig. 4 and Supplementary Fig. S5).

### Determination of the cut-off value and its application in a rapid detection test

A receiver operating characteristic (ROC) curve was drawn for the percent hydrolysis of 148 strains at different incubation times (30, 60, 120, and 180 min) to distinguish between CPE and non-CPE strains (Supplementary Fig. S6). The average of two threshold values obtained from the highest Youden’s J statistic was used to estimate an optimal cut-off value for percent hydrolysis. The calculated cut-off values were as follows: 9.43 (30 min), 13.89 (60 min), 23.90 (120 min), and 31.87 (180 min). The cut-off value of 9.43 after a 30-min incubation had 98.23% sensitivity and 100% specificity. Two OXA-232 producers were mistakenly recognized as non-CPE strains at the 30-min time point. However, the percent hydrolysis of these strains subsequently increased (Fig. 3b), and 100% sensitivity and specificity were obtained after 60–180-min incubations (Supplementary Table S4). The minimum number of bacterial cells required for the detection of CPE was approximately 8.3 × 10^4^ CFU (or 8.3 × 10^5^ CFU/ml) and 9.2 × 10^7^ CFU (or 9.2 × 10^8^ CFU/ml) per reaction mixture for the strain exhibiting highest and lowest %hydrolysis, respectively.

### Quantification of bacterial hydrolytic activity to meropenem

Other carbapenems are also known to show similar absorption spectra and peak absorbance to that of imipenem (Supplementary Fig. S7). Considering a possible utilization of other carbapenems in our method, %hydrolysis to meropenem was measured for the same 148 strains with the same calculation formula as that for imipenem. The IMP-, KPC-, and NDM-producers exhibited sufficient hydrolytic activity to differentiate them from non-CPE strains (Supplementary Fig. S8). However, several OXA-producers showed negligible or no %hydrolysis, and they could not be separated from non-CPE strains even after a 180-min incubation.

### Comparison with the Carba NP test

The Carba NP test was performed for the same 148 strains to compare the methods as rapid detection tests. After a 30-min incubation, Carba NP displayed 85.84% sensitivity and 100% specificity, leaving 16 CPE strains (11 OXA-181 and five OXA-232 producers) undetected. After a 120-min incubation, which is the maximum incubation time recommended by the manufacturer, the Carba NP test displayed 95.58% sensitivity and 94.29% specificity (Supplementary Table S5 online). Five strains (three OXA-232 and two OXA-181 producers) could not be detected as CPE strains. Two non-CPE strains displayed a change in the colour of Phenol Red after a 120-min incubation and were mistakenly recognized as CPE strains.

### Confirmation of carbapenemase production by the Carbapenem Inactivation Method (CIM) and modified CIM (mCIM)

The presence or absence of carbapenemase production was examined for CPE and non-CPE strains. All CPE strains except for one IMP-producer strain exhibited positive results on CIM. The IMP-producer also exhibited positive results when subjected to mCIM. Consequently, all CPE strains were confirmed to produce carbapenemase. However, all non-CPE strains showed negative results on CIM.

## Discussion

Spectrophotometry is one of the most reliable methods for measuring the hydrolytic activity of carbapenemases^7,10,11^. Our method is distinct from the previous method in that whole bacterial cells can be directly used to measure carbapenemase activity, thereby achieving a rapid CPE detection system.

Previously, carbapenemase needed to be extracted from bacterial cells to minimize the absorption by various biomolecules (e.g. nucleic acids, proteins, and chemicals), which interfere with imipenem absorption in the UV region^10^. The procedure was time-consuming, taking 12–24 hours from the inoculation of bacterial colonies. In addition, it comprised numerous steps and required expertise. Accordingly, the UV spectrophotometric method has not been adopted for the first rapid differentiation between CPE and non-CPE strains^11^.

In this study, we devised a method to directly apply whole bacterial cells for UV spectrophotometric measurement of hydrolytic activity. The direct application of bacterial cells enabled reductions in sample preparation time to less than 10 min, and the method could differentiate CPE from non-CPE strains as early as a 30-min incubation. Our system expedited the previous UV spectrophotometric method to be a possible candidate for the first screening of CPE, which was previously considered a role of the Carba NP test^11^.

OXA-type carbapenemases exhibit relatively weak hydrolytic activity and are difficult targets of phenotypic detection systems^6,7,9^. The RAPIDEC^®^ CARBA NP test evaluated in this study could not detect the hydrolytic activity of some OXA producers with a maximum incubation time of 120 min. Our method also showed lower rates of imipenem hydrolysis by OXA producers in comparison with other types of carbapenemase producers (Fig. 3a), and it mistakenly recognized 2 OXA producers as non-CPE strains, based on the cut-off value of 9.43 after a 30-min incubation. However, our method yielded an increase in imipenem hydrolysis with extended incubation times, and the 2 OXA-producing strains were accurately detected as CPE after 60–180 min. The improved sensitivity in detecting OXA-type producers is an advantage of this method.

Considering a potential application of other carbapenems in the detection system, we also examined the %hydrolysis to meropenem. However, several OXA-producers showed negligible or no %hydrolysis to meropenem with unknown reason. They did not increase the %hydrolysis with an extended incubation time, which was confirmed in the imipenem hydrolysis. Therefore, imipenem was considered a suitable indicator of carbapenemase activity in our system, although other carbapenems may have to be examined to identify the most optimal agent.

We obtained consistent results upon CIM and mCIM. CIM is one of the standard assays to detect carbapenemase production^16^, and mCIM is a technique recommended by Clinical and Laboratory Standards Institute (CLSI) working group^17^. Although these methods take 8–22 hours to perform and are not considered rapid detection tests, they are very reliable methods to confirm carbapenemase production. Furthermore, the consistent results with our method suggests that the accuracy of this method to detect carbapenemase production is high.

The MICs of imipenem and the associated hydrolytic activities did not correlate with each other (Fig. 4). One reason for this discrepancy is probably the relatively slow hydrolysis of imipenem by OXA-type carbapenemase producers (Supplementary Fig. S4). Although 75% of them showed MICs ≥ 4 μg/ml (Supplementary Fig. S5), even these strains exhibited lower activities than that of other CPE strains with similar MICs by the end of a 120-min incubation (Supplementary Fig. S9). Furthermore, the dissociation between MICs and the percent hydrolysis of IMP-type carbapenemase producers may constitute another reason. IMP producers are known to exhibit relatively low MICs for imipenem^18–20^. In accordance with previous reports, 85% of IMP producers in this study showed imipenem MICs ≤ 1 μg/ml (Supplementary Fig. S5). Despite their low MICs, they exhibited higher hydrolytic activities than OXA producers with our spectrophotometric method (Fig. 3a). Differing enzyme stabilities and expression levels can contribute to these results. In addition, the outer membrane permeability of imipenem may differ among CPE strains and contribute to the discrepancy. Porins are reported to constitute the primary route of periplasmic entry of imipenem^21,22^, and varying expression levels or porin mutations can affect the results. A strain with reduced permeability will hydrolyse lesser imipenem in our system, while still exhibiting a high MIC. Although the mechanisms underlying this discrepancy are unknown, the established system successfully detected CPE strains with low imipenem MICs, resulting in high sensitivity.

This study has several limitations. Although the dual-wavelength spectrophotometric method is simple and reliable, the system requires a spectrophotometer, which may not be available in some hospital-based clinical microbiology laboratories. Furthermore, absorbance can vary depending on the instrument and measurement settings. Hence, equation (1) and cut-off value must be reconfigured and recalculated in individual laboratories. Therefore, we intend on developing a portable instrument based on our system, which should enable successful applications for clinical use in medical facilities. In addition, the sample size was small; hence, future studies with larger sample sizes are required, and a prospective study should be performed to calculate the sensitivity and specificity more precisely. These parameters can vary if a prospective study includes non-carbapenemase-producing CRE, such as extended spectrum β-lactamases, and/or AmpC β-lactamase producers with decreased membrane permeability^23^, which were not included in this study.

In summary, we developed a new and simple method to quantify the hydrolytic activity of CPE based on the use of a UV–VIS spectrophotometer. Generalization of the system by developing a companion apparatus will lead to the rapid detection of CPE in the clinical setting and support treatment decisions and infection-control measures.

## Methods

### Bacterial strains and cultures

The bacterial strains used in this study are listed in Supplementary Tables S1 and S3. Ten reference strains were purchased from the American Type Culture Collection (ATCC, Manassas, VA, USA). The other 138 strains were clinical isolates collected from patients in multiple hospitals in Japan, Myanmar, Singapore, and Thailand. API 20E strips (SYSMEX bioMérieux, Marcy-l′Étoile, France) were used for bacterial identification. Antibiotic-susceptibility testing and MIC measurements were performed for all strains, using the microdilution method with Dry plates (EIKEN, Tokyo, Japan). Ertapenem MICs were examined for the strains from Thailand, using the automated Sensititre system (Trek, East Grinstead, UK).

CRE were defined as *Enterobacteriaceae* that acquired resistance to at least one of the carbapenems tested, including meropenem (≥4 μg/ml), imipenem (≥4 μg/ml), doripenem (≥4 μg/ml), ertapenem (≥2 μg/ml), and panipenem (≥4 μg/ml).

CPE were defined as CRE harbouring the carbapenemase gene. The presence or absence of carbapenemase genes was examined via Multiplex PCR method^24^, PCR-dipstick chromatography^25^, or whole-genome sequencing on a PacBio RSII (Pacific Bioscience, Menlo Park, CA, USA), MiSeq (Illumina, San Diego, CA, USA), or HiSeq 3000 (Illumina) instrument.

Strains were cultured on Brain Heart Infusion (BHI) agar (Becton, Dickinson and Company, Franklin Lakes, NJ, USA) and Mueller Hinton E (MHE) agar (SYSMEX bioMérieux) for spectrophotometric measurements of imipenem hydrolysis and Carba NP testing, respectively.

### Spectrophotometric analysis of carbapenems

The absorbance of carbapenems, including imipenem (Wako Pure Chemical Industries, Osaka, Japan), meropenem (Tokyo Chemical Industry, Tokyo, Japan), doripenem, biapenem, and ertapenem (Merck, Kenilworth, NJ, USA), was measured using a Corona plate reader SH-9000 (Hitachi, Tokyo, Japan) at room temperature (approximately 25 °C) in a UV-transparent 96-well plate (Zell-kontakt, Nörten-Hardenberg, Germany). Measurements with bandwidths of 1 nm and 5 nm were implemented for analysing absorption spectra.

### Imipenem hydrolysis by bacterial cells

Fifty microliters of imipenem solution (50 μg/ml) or PBS was mixed with 50 μl of each bacterial suspension (6.0 × 10^8^ CFU/ml). The mixture was incubated at 37 °C for 60 min, and the absorbance was measured between 220 nm and 400 nm. *Klebsiella pneumoniae* ATCC 4352 and ATCC BAA-2470 were used as representative non-CPE and CPE strains, respectively.

### Estimation of background absorbance

Fifteen representative strains were selected for this study (Supplementary Table S1). A bacterial suspension of each strain was prepared at an optical density at 600 nm of 3.0, and two-fold serial dilutions (up to a 1:64 dilution) were performed to generate bacterial suspensions at eight different concentrations. The control comprised PBS without bacteria. Fifty microliters of the bacterial suspension was mixed with 50 μl of imipenem solution (50 μg/ml) or PBS. Absorbance was measured at 297 nm and 350 nm wavelength after incubation at 37 °C for 30, 60, 120, or 180 min. Triplicate samples were prepared, and each measurement was independently performed at least three times.

### Quantification of bacterial hydrolytic activities

The 148 strains tested in this study (Supplementary Table S3) were cultured on agar plates overnight, and several colonies for each strain were suspended in 100 μl of imipenem solution (25 μg/ml) to yield turbidity of McFarland standard number 5 (SYSMEX bioMerieux). The turbidity corresponds to a bacterial concentration of approximately 1.5 × 10^9^ CFUs/ml. After incubation at 37 °C for 30, 60, 120, or 180 min, the absorbance of each reaction mixture at 297 nm and 350 nm was measured to quantify bacterial hydrolytic activity. Percent hydrolysis was calculated as follows:

$${\rm{100}}\times (1-[{\rm{gAbs}}.{\rm{297}}\,{\rm{nm}}-{\rm{eAbs}}.{\rm{297}}\,\mathrm{nm}]/[{\rm{Abs}}.\,{\rm{of}}\,{\rm{original}}\,{\rm{amount}}\,{\rm{of}}\,{\rm{imipenem}}-{\rm{Abs}}{\rm{.}}\,{\rm{of}}\,{\rm{PBS}}]){\rm{.}}$$

To determine the minimum number of bacterial cells required for optimal functioning of our detection system, CPE strains with highest and lowest %hydrolysis were selected for the analysis. Serially diluted bacterial suspensions were mixed with imipenem solution, and %hydrolysis at 180 min were measured and compared with corresponding bacterial concentrations. Minimum number of bacterial cells required to achieve the cut-off value at 180 min incubation was considered the detection limit.

The quantification of bacterial hydrolytic activity to meropenem was implemented similarly, as described above.

### Carba NP test

Carbapenemase production was examined using the RAPIDEC^®^ CARBA NP (SYSMEX bioMerieux) in accordance with the manufacturer’s instructions. Briefly, bacterial suspensions were added to a mixture containing substrate and pH indicator. Readings were obtained visually, and the colour was compared with that of a control well after a 30 min, 60 min, or 120 min of incubation. Three investigators, who were blinded to genus, species, and genotypes of the strains, obtained the readings. Inconsistent results were resolved by the majority decision of the investigators.

### Carbapenem Inactivation Method (CIM)

The CIM was performed for all CPE and non-CPE strains as described previously^16^. The modified CIM^17^ was also implemented for a strain, wherein a slight inhibition zone around the meropenem disk was observed and carbapenemase production could not be confirmed via CIM.

### Statistical analysis

All statistical analysis was performed using GraphPad Prism 5 (GraphPad software, La Jolla, CA, USA) and EZR statistical software^26^. An alpha level of 0.05 was used for all tests. Global testing was performed to confirm statistical differences among all groups. Pair-wise comparison was performed only when statistical significance was observed upon global analysis. Non-parametric tests were used for all comparisons except for the analysis in Supplementary Fig. S2, where parametric paired *t*-testing was used, based on the normally distributed data. Other information related to statistical analysis is provided in the Results section and figure legends.

## Electronic supplementary material

Supplementary Information

## Data Availability

The datasets generated and/or analysed during the current study are available from the corresponding author upon reasonable request.

## References

[1] Banerjee R, Humphries R (2017). Clinical and laboratory considerations for the rapid detection of carbapenem-resistant Enterobacteriaceae. Virulence.

[2] Tamma PD (2017). Comparing the outcomes of patients with carbapenemase-producing and non-carbapenemase-producing carbapenem-resistant Enterobacteriaceae bacteremia. Clin. Infect. Dis..

[3] Temkin E, Adler A, Lerner A, Carmeli Y (2014). Carbapenem-resistant Enterobacteriaceae: biology, epidemiology, and management. Ann. N. Y. Acad. Sci..

[4] Nordmann P, Naas T, Poirel L (2011). Global spread of carbapenemase-producing Enterobacteriaceae. Emerg. Infect. Dis..

[5] Dortet L (2015). Evaluation of the RAPIDEC(R) CARBA NP, the Rapid CARB Screen(R) and the Carba NP test for biochemical detection of carbapenemase-producing Enterobacteriaceae. J. Antimicrob. Chemother..

[6] Morey, K. E. *et al*. Evaluation of the Carba NP test in Oregon, 2013. *Antimicrob. Agents Chemother*. **61**, 10.1128/AAC.03005-15 (2017). 10.1128/AAC.03005-15 PMC5192107 27795386

[7] Osterblad M, Hakanen AJ, Jalava J (2014). Evaluation of the Carba NP test for carbapenemase detection. Antimicrob. Agents Chemother..

[8] Poirel L, Nordmann P (2015). Rapidec Carba NP test for rapid detection of carbapenemase producers. J. Clin. Microbiol..

[9] Tijet N, Boyd D, Patel SN, Mulvey MR, Melano RG (2013). Evaluation of the Carba NP test for rapid detection of carbapenemase-producing Enterobacteriaceae and Pseudomonas aeruginosa. Antimicrob. Agents Chemother..

[10] Bernabeu S, Poirel L, Nordmann P (2012). Spectrophotometry-based detection of carbapenemase producers among Enterobacteriaceae. Diagn. Microbiol. Infect. Dis..

[11] Dortet L, Brechard L, Cuzon G, Poirel L, Nordmann P (2014). Strategy for rapid detection of carbapenemase-producing Enterobacteriaceae. Antimicrob. Agents Chemother..

[12] Girlich D, Poirel L, Nordmann P (2012). Diversity of naturally occurring Ambler class B metallo-beta-lactamases in Erythrobacter spp. J. Antimicrob. Chemother..

[13] Gudeta DD (2016). Chromobacterium spp. harbour Ambler class A beta-lactamases showing high identity with KPC. J. Antimicrob. Chemother..

[14] Osterblad M (2012). Carbapenemase-producing Enterobacteriaceae in Finland: the first years (2008–11). J. Antimicrob. Chemother..

[15] Roh KH (2010). New cfiA variant and novel insertion sequence elements in carbapenem-resistant Bacteroides fragilis isolates from Korea. Diagn. Microbiol. Infect. Dis..

[16] van der Zwaluw K (2015). The carbapenem inactivation method (CIM), a simple and low-cost alternative for the Carba NP test to assess phenotypic carbapenemase activity in gram-negative rods. PLoS One.

[17] Pierce VM (2017). Modified Carbapenem Inactivation Method for Phenotypic Detection of Carbapenemase Production among Enterobacteriaceae. J. Clin. Microbiol..

[18] Hayakawa K (2014). Molecular and epidemiological characterization of IMP-type metallo-beta-lactamase-producing Enterobacter cloacae in a Large tertiary care hospital in Japan. Antimicrob. Agents Chemother..

[19] Yano H (2001). Plasmid-encoded metallo-beta-lactamase (IMP-6) conferring resistance to carbapenems, especially meropenem. Antimicrob. Agents Chemother..

[20] Yano H (2012). High frequency of IMP-6 among clinical isolates of metallo-beta-lactamase-producing Escherichia coli in Japan. Antimicrob. Agents Chemother..

[21] Bajaj H (2016). Molecular basis of filtering carbapenems by porins from beta-lactam-resistant clinical strains of Escherichia coli. J. Biol. Chem..

[22] Tran QT (2014). Structure-kinetic relationship of carbapenem antibacterials permeating through E. coli OmpC porin. Proteins.

[23] Tamma PD (2017). Comparison of 11 phenotypic assays for accurate detection of carbapenemase-producing Enterobacteriaceae. J. Clin. Microbiol..

[24] Dallenne C, Da Costa A, Decre D, Favier C, Arlet G (2010). Development of a set of multiplex PCR assays for the detection of genes encoding important beta-lactamases in Enterobacteriaceae. J. Antimicrob. Chemother..

[25] Shanmugakani, R. K. *et al*. PCR-dipstick chromatography for differential detection of carbapenemase genes directly in stool specimens. *Antimicrob. Agents Chemother*. **61**, 10.1128/AAC.00067-17 (2017). 10.1128/AAC.00067-17 PMC5444138 28373197

[26] Kanda Y (2013). Investigation of the freely available easy-to-use software ‘EZR’ for medical statistics. Bone Marrow Transplant..

