# Supplementary material for: Establishment of a dual-wavelength spectrophotometric method for analysing and detecting carbapenemase-producing Enterobacteriaceae
Source: Sci Rep. 2018 Oct 24;8:15689. doi: 10.1038/s41598-018-33883-0 (PMC6200816; doi:10.1038/s41598-018-33883-0)

**Establishment of a dual-wavelength spectrophotometric method for analysing and detecting carbapenemase-producing *Enterobacteriaceae***

**Authors**

Dan Takeuchi, Yukihiro Akeda, Yo Sugawara, Noriko Sakamoto, Norihisa Yamamoto, Rathina Kumar Shanmugakani, Takuma Ishihara, Ayumi Shintani, Kazunori Tomono, and Shigeyuki Hamada


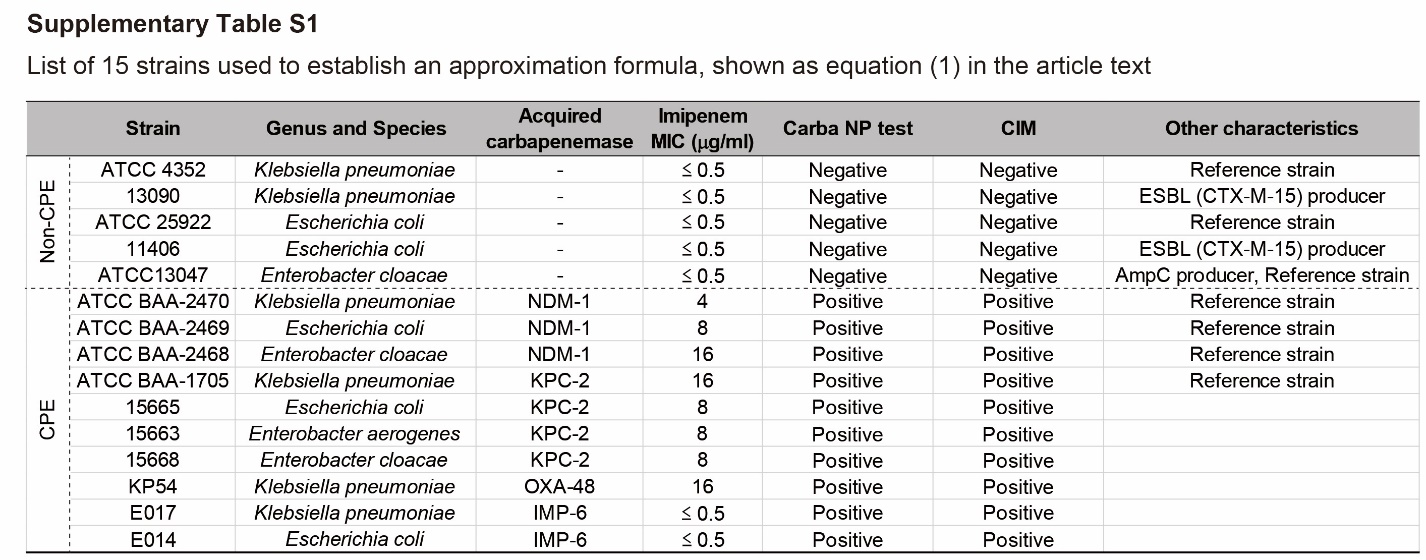


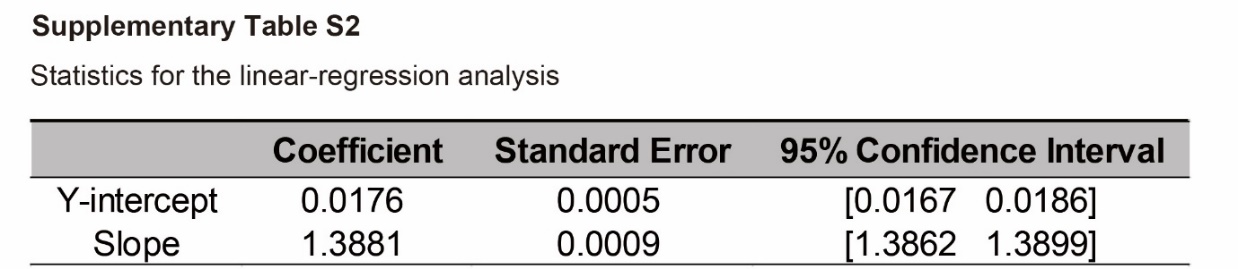


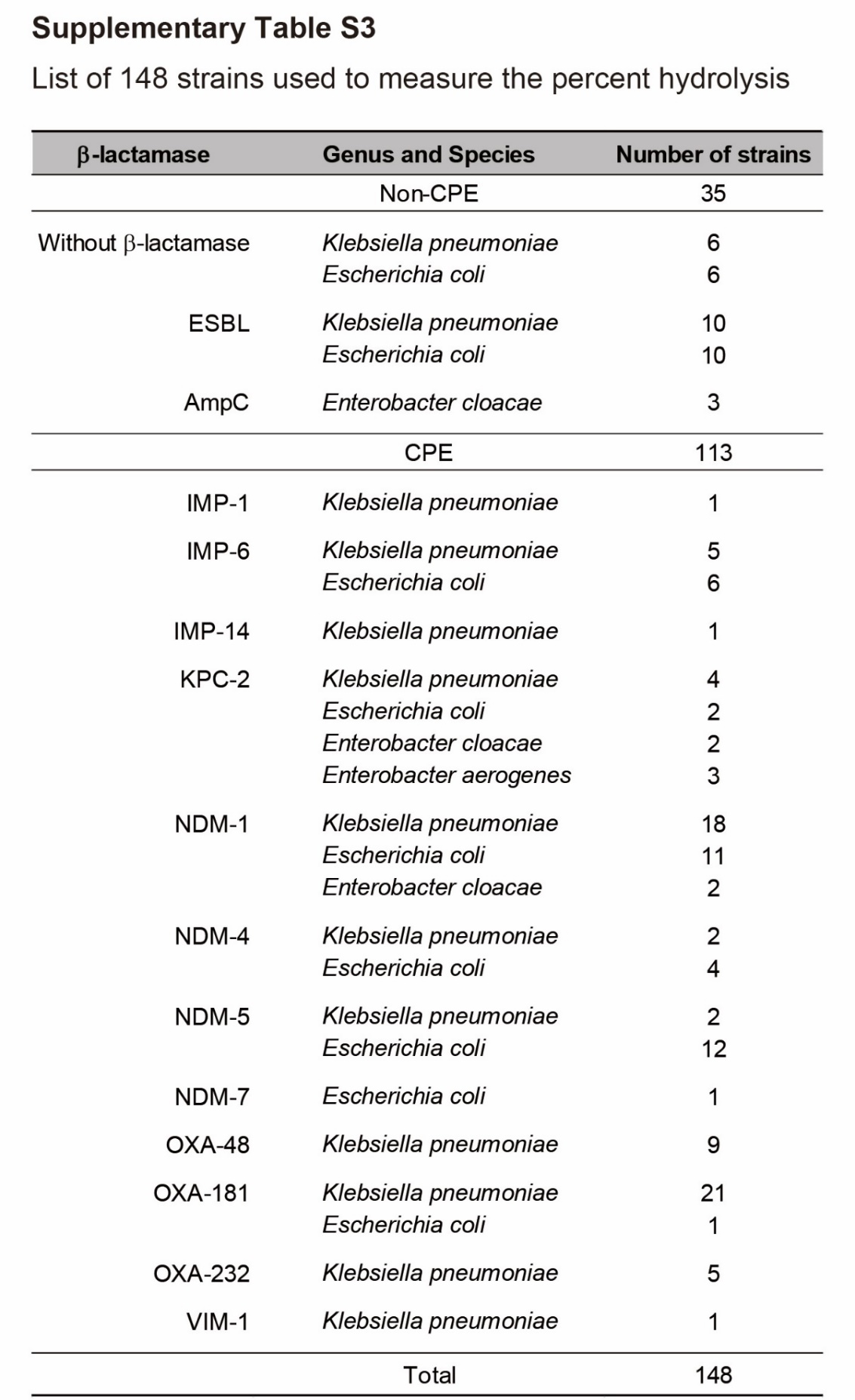


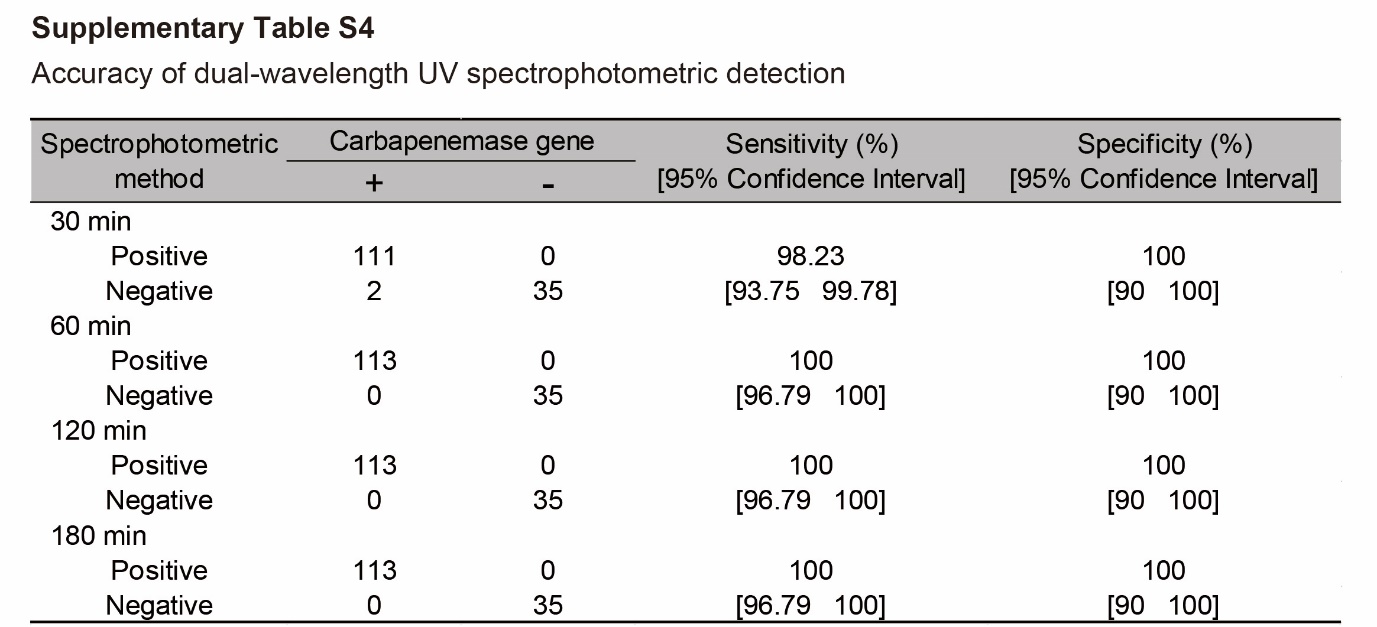


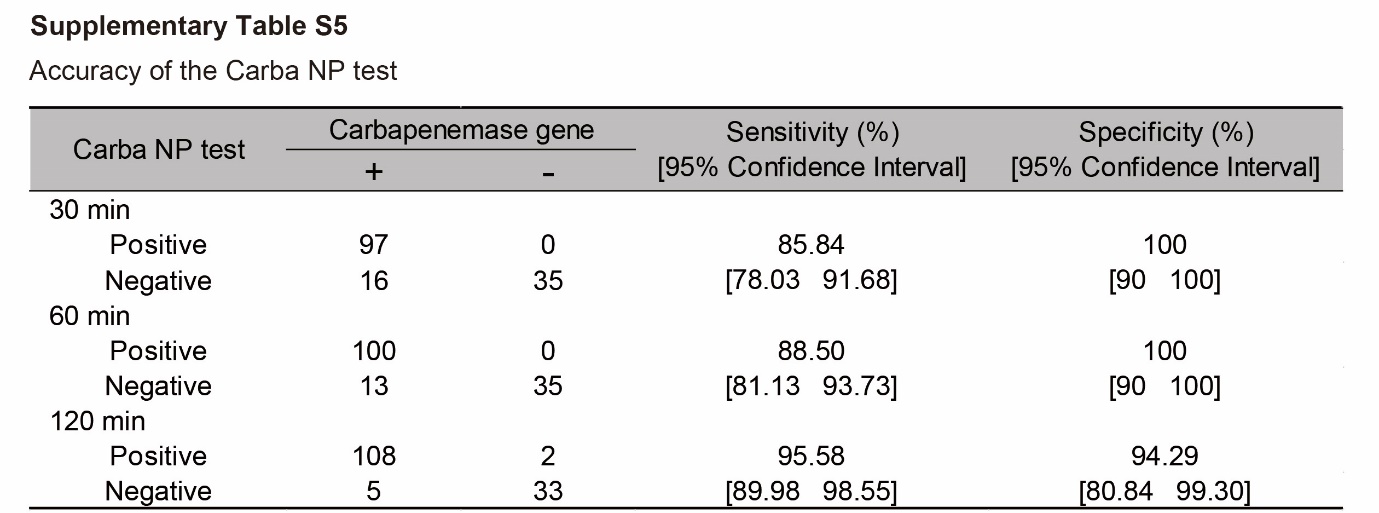


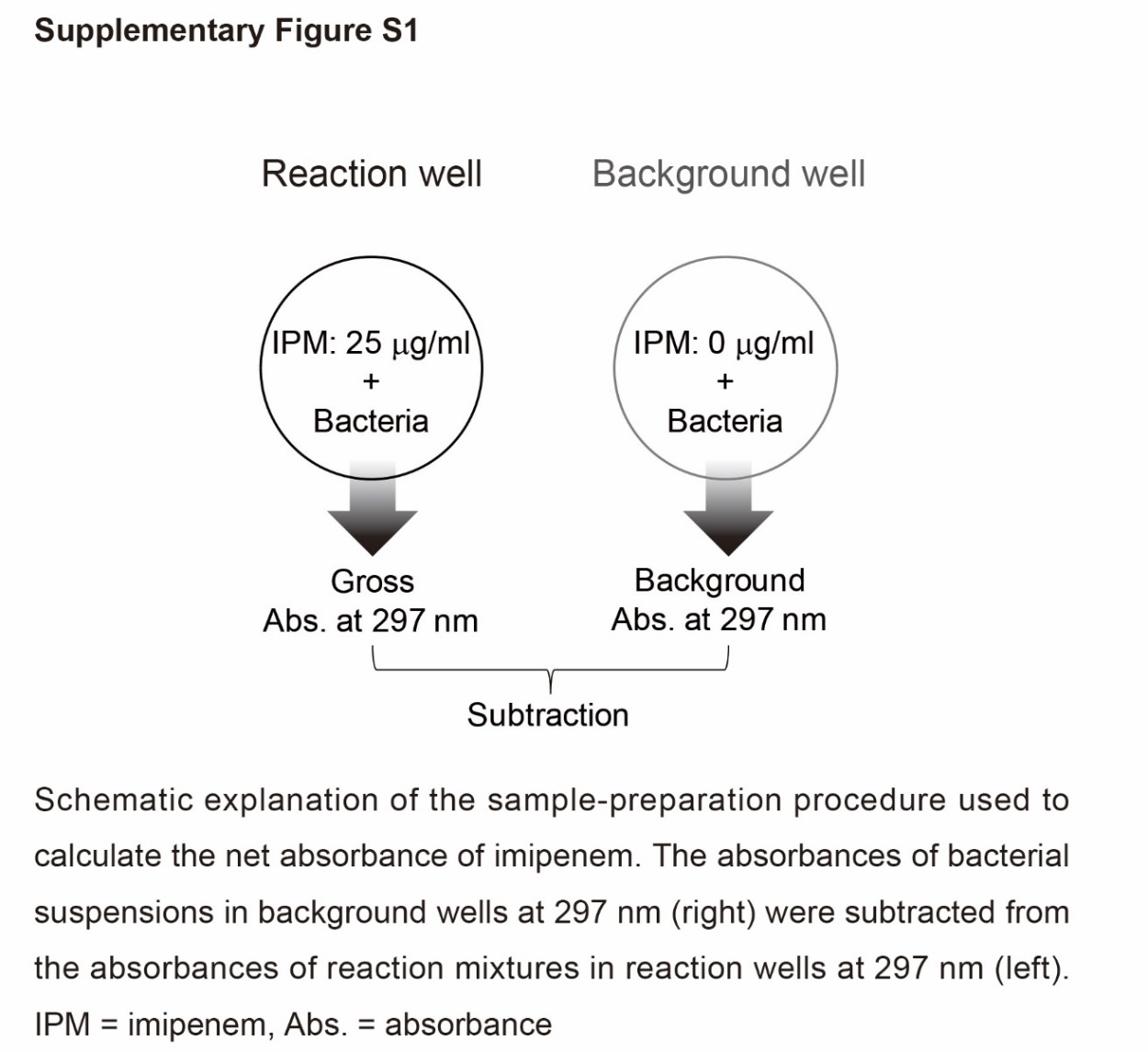


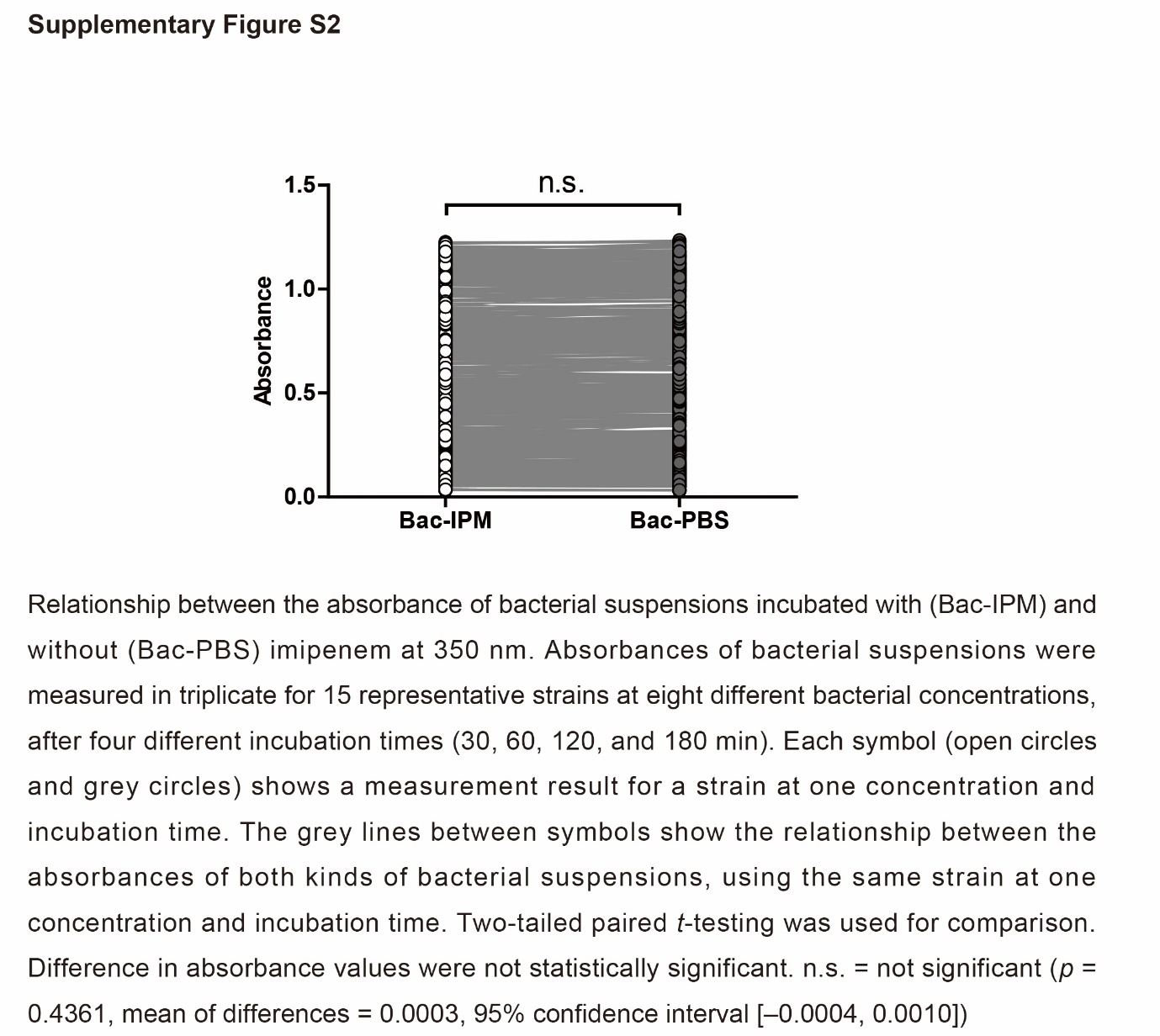


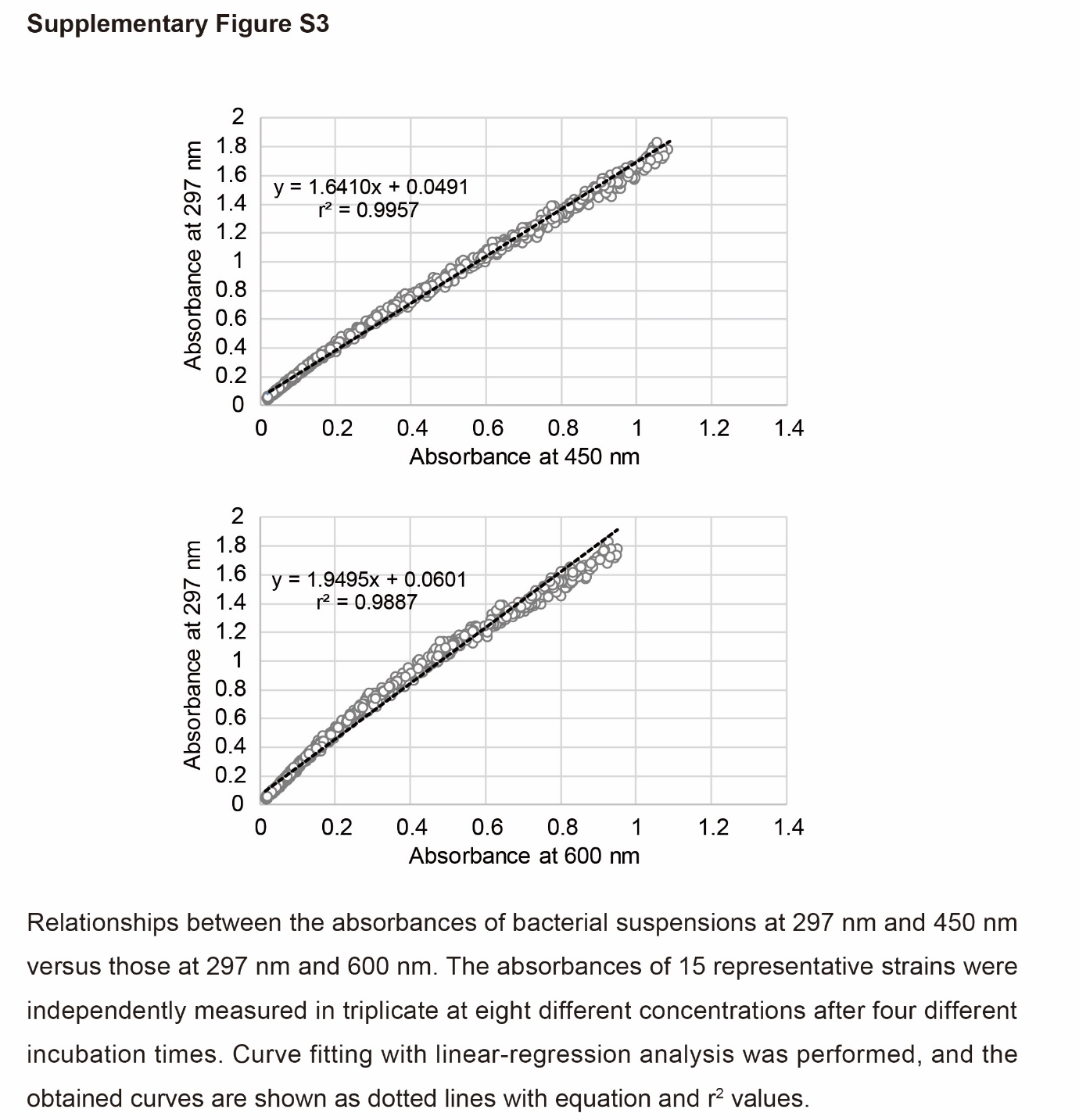


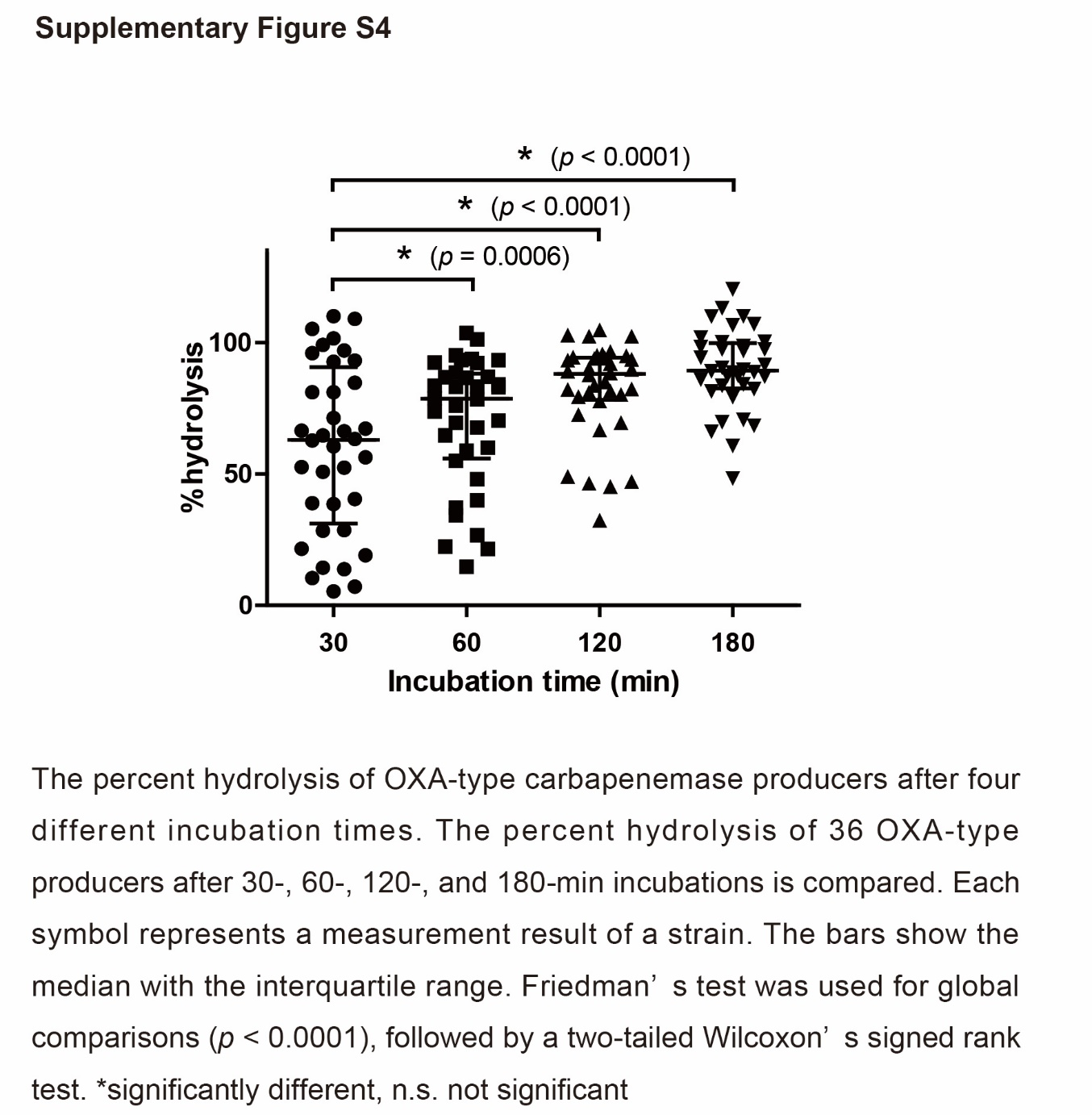


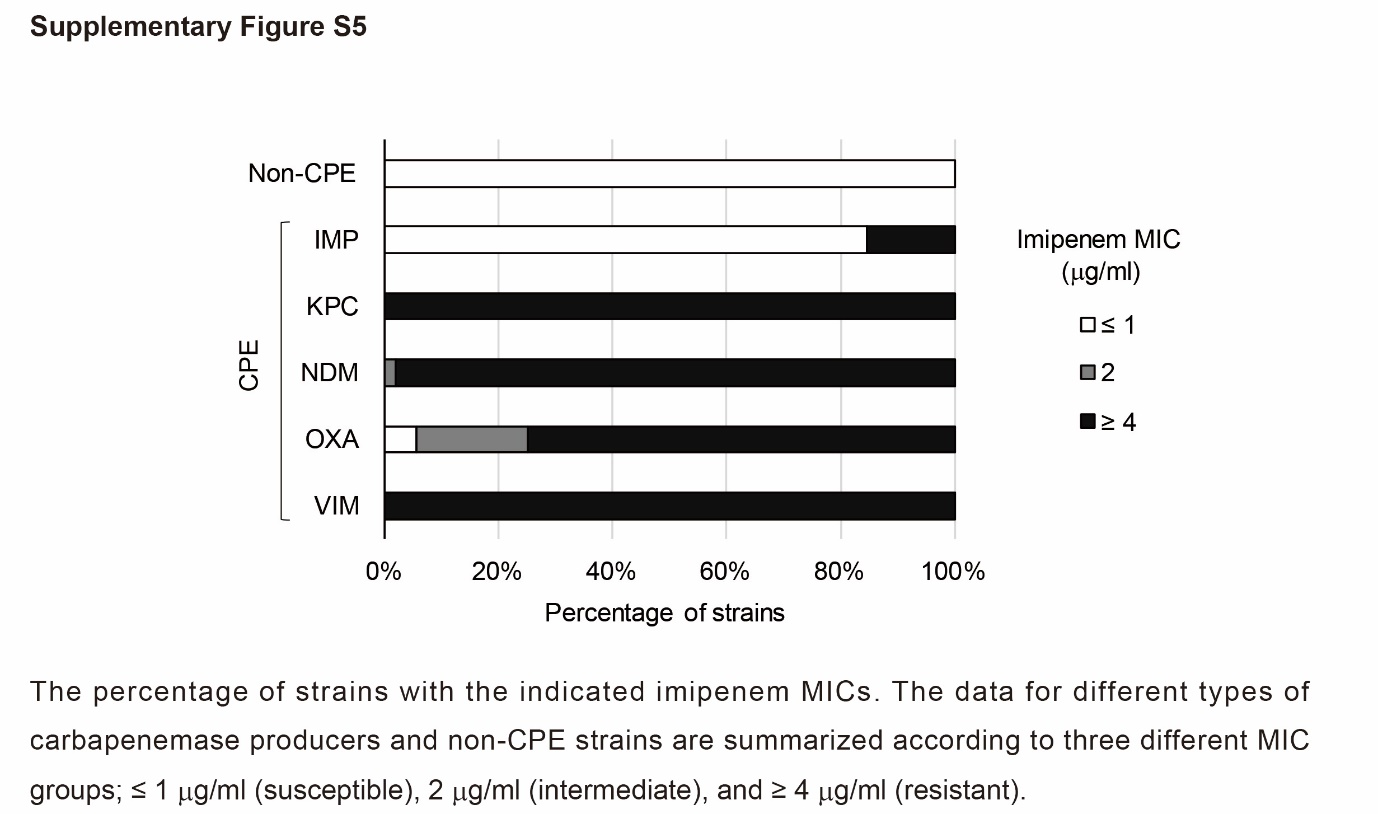


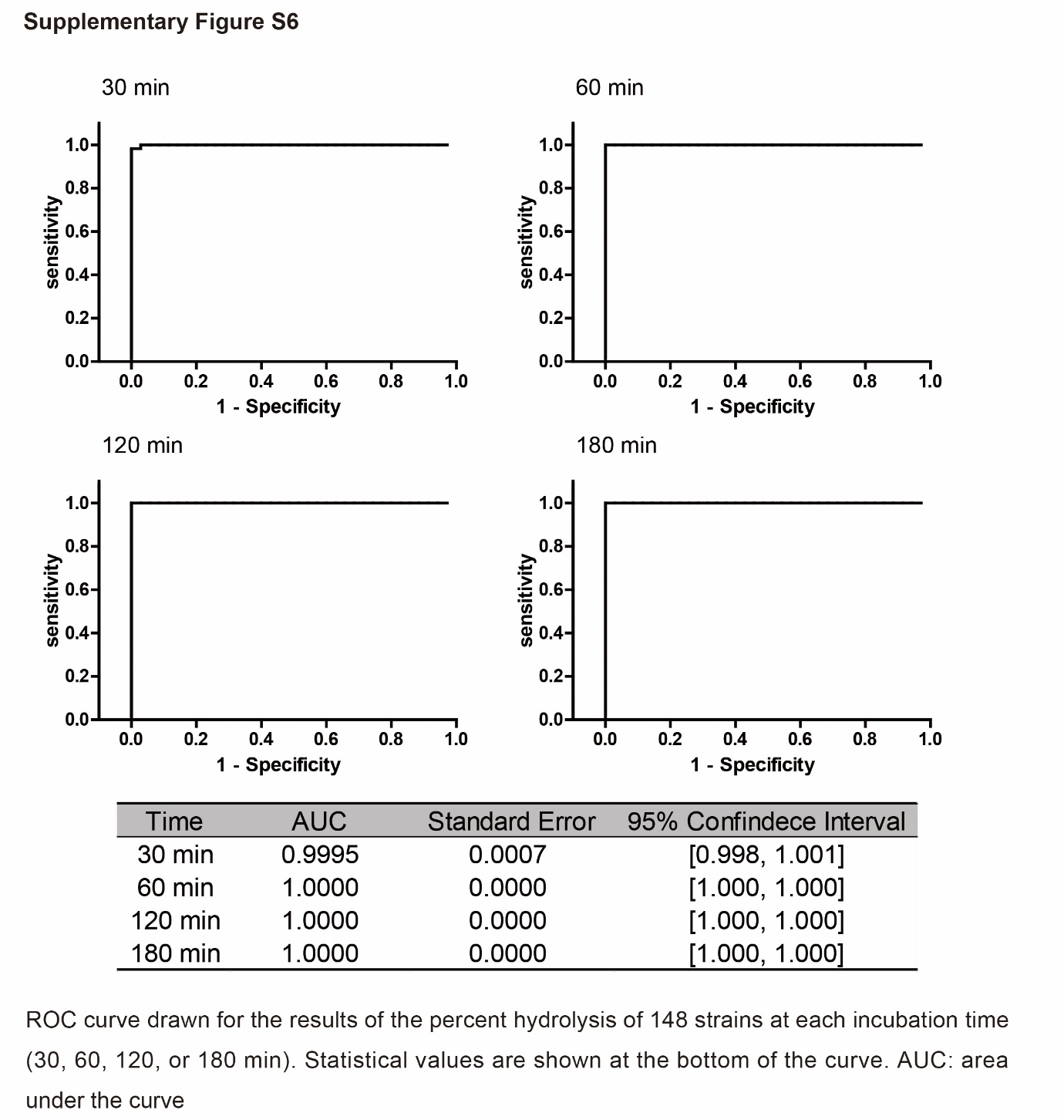


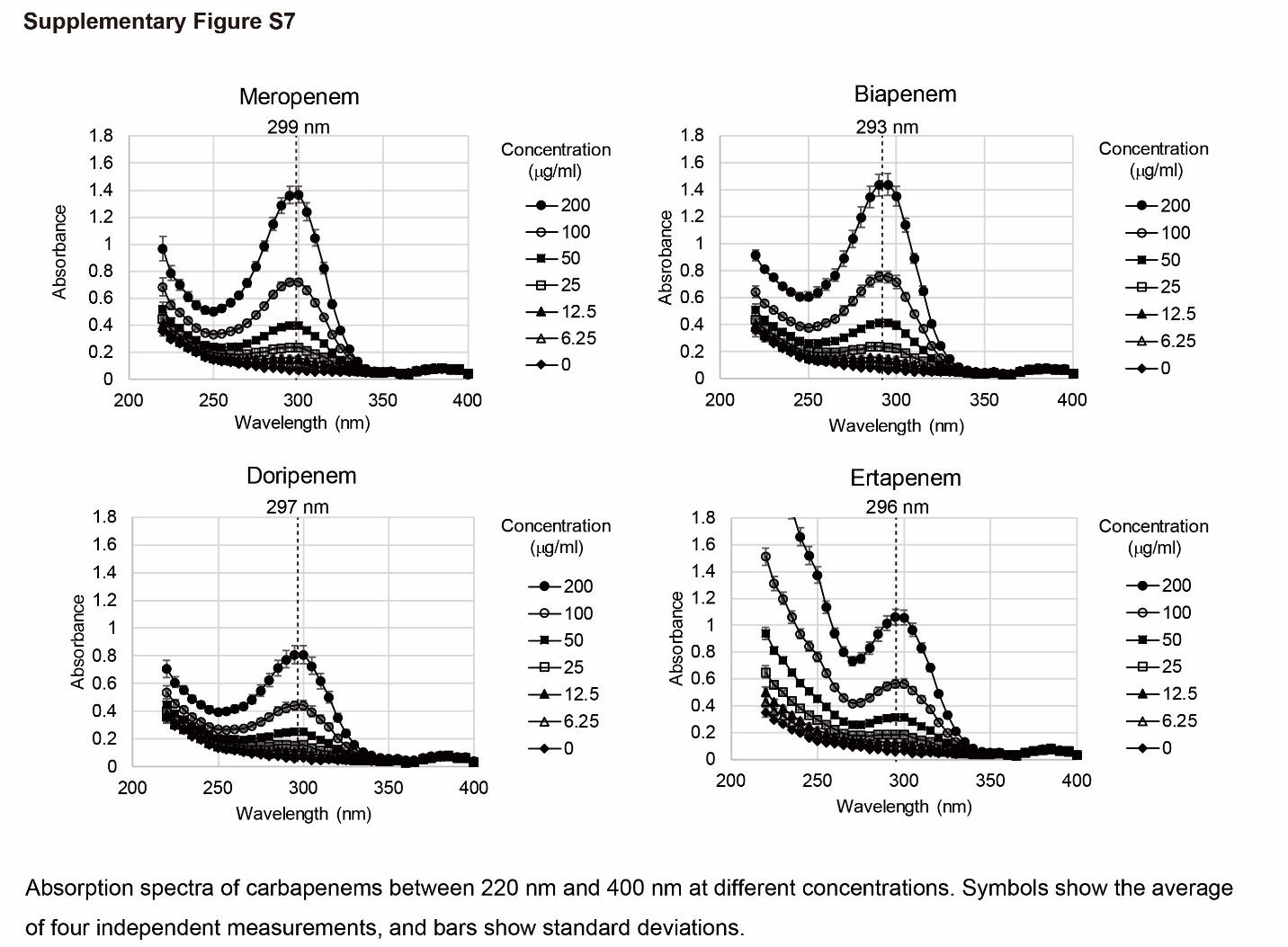


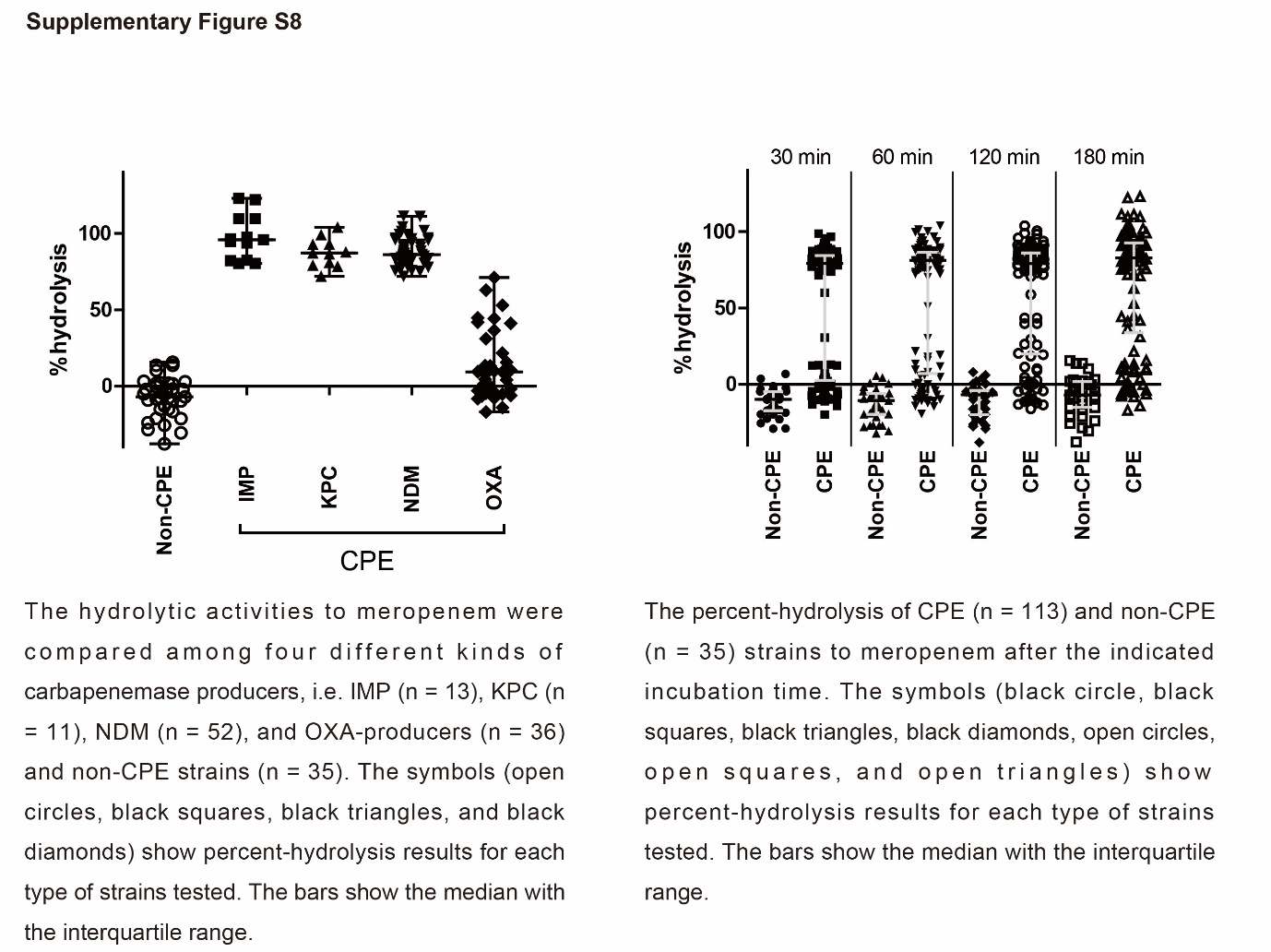


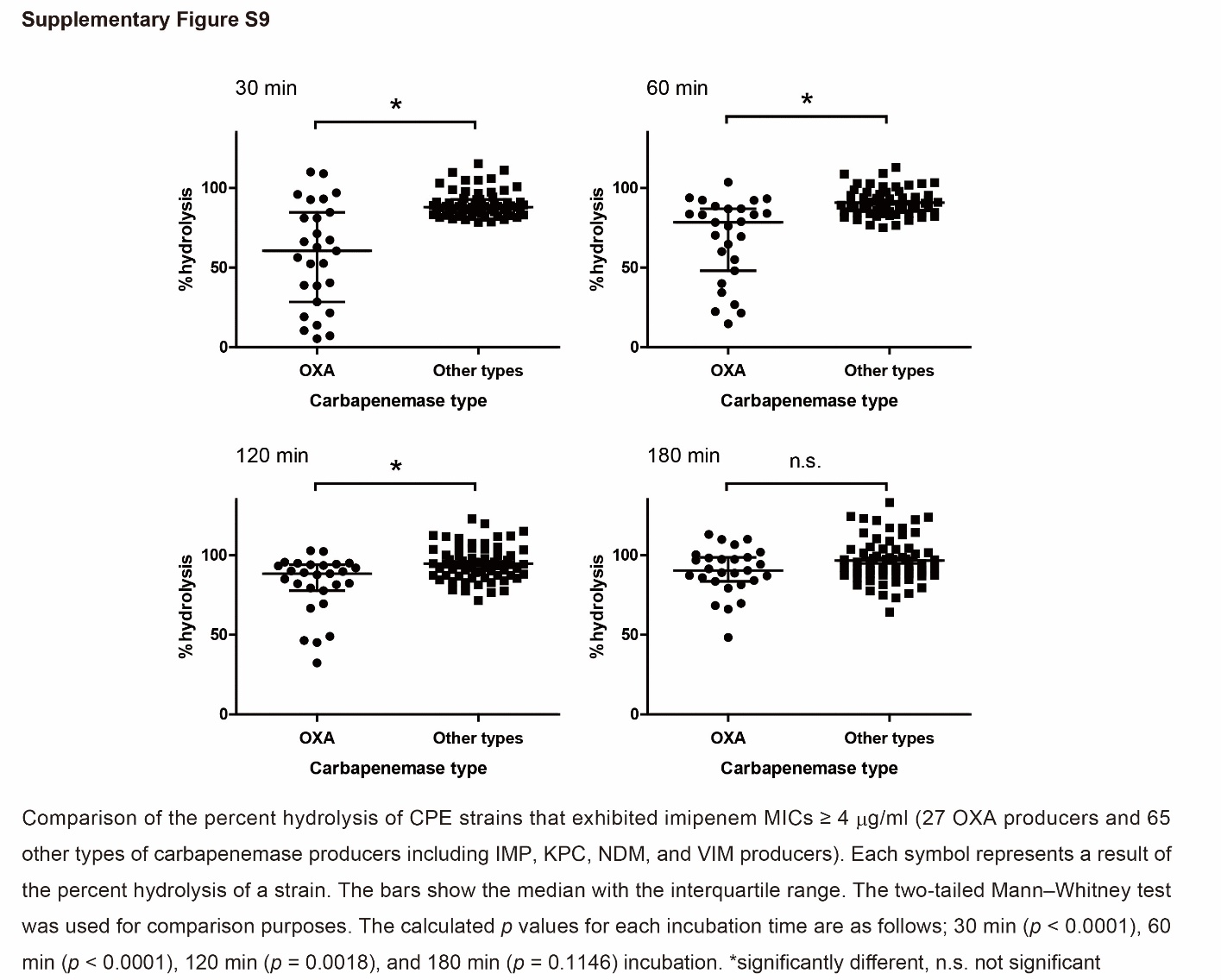

Supplement: Supplementary file 1 — Supplementary Information [file 41598_2018_33883_MOESM1_ESM.docx]
